# Dystrophin isoform deficiency and upper‐limb and respiratory function in Duchenne muscular dystrophy

**DOI:** 10.1111/dmcn.16282

**Published:** 2025-03-14

**Authors:** Mary Chesshyre, Deborah Ridout, Georgia Stimpson, Valeria Ricotti, Silvana De Lucia, Erik H. Niks, Volker Straub, Laurent Servais, Jean‐Yves Hogrel, Giovanni Baranello, Adnan Manzur, UK NorthStar Clinical Network, Francesco Muntoni, the iMDEX Network, Joana Domingos, Victoria Selby, Amy Wolfe, Lianne Abbott, Efthymia Panagiotopoulou, Mario Iodice, Maria Ash, Imelda de Groot, Merel Jansen, Maaike Pelsma, Marian Bobbert, Menno Van Der Holst, Yvonne D. Krom, Marjolein J. van Heur‐Neuman, Thomas Voit, Valérie Decostre, Stéphanie Gilabert, Michela Guglieri, Alexander Murphy, Anna Mayhew, Mariacristina Scoto, Anna Sarkozy, Pinki Munot, Stephanie Robb, Elaine Chan, V Robinson, W Girshab, V Crook, E Milev, L Abbott, A Wolfe, E O’Reilly, J Watts‐When, N Burnett, R Thomas, R Terespolsky, O Martinaeu, J Longatto, C Bettolo, M Guglieri, J Diaz‐Manera, G Tasca, M Elseed, R Muni‐Lofra, M James, D Moat, J Sodhi, K Wong, E Robinson, E Groves, R Rabb, H McMurchie, H Chase, Tracey Willis, C Rylance, N Birchall, E Wright, A Childs, K Pysden, C Martos, D Roberts, L. Pallant, S Walker, A Henderson, R Madhu, R Karuvattil, Y Balla, S Gregson, S Clark, E Wraige, H Jungbluth, V Gowda, M Vanegas, J. Sheehan, A Schofield, C Smith, I Hughes, E Whitehouse, S Warner, E Reading, N Emery, J Moustoukas, K Strachan, M Ong, M Atherton, N Mills, S Sanchez Marco, A Saxena, K Skone, J TeWaterNaude, H Davis, C Wood, A Majumdar, A Murugan, I Guarino, R Tomlinson, H Jarvis, L Wills, C Frimpong, J Watson, G Cobb, G Robertson, P Brink, J Burslem, C Adams, J Wong, S Joseph, I Horrocks, J Dunne, M DiMarco, S Brown, S McKenzie, K Torne, R Mohamed, V Velmurugan, M Prasad, S Sedehizadeh, A Schugal, R Keetley, S Williamson, K Payne, E Dowling, P Fenty, C de Goede, A Parkes, K Baxter, M Illingworth, N Bhangu, S Geary, J Palmer, K Shill, S Tirupathi, A Shah, D O’Donogue, J McVeigh, J McFetridge, G Nicfhirleinn, H Beattie, T Leyland, K Stevenson, N Hussain, D Baskaran, Z Lambat, R Sullivan, L Locke, G Ambegaonkar, D Krishnakumar, J Taylor, J Moores, E Stephen, J Tewnion, S Ramdas, M Sa, A Skippen, M Khries, C Lilien, H Ramjattan, F Taylor, H English, K Stewart, F Flint, E Bartram, R Noble

**Affiliations:** ^1^ Dubowitz Neuromuscular Centre UCL Great Ormond Street Institute of Child Health and Great Ormond Street Hospital London UK; ^2^ Population, Policy and Practice Research and Teaching Department UCL GOS Institute of Child Health London UK; ^3^ NIHR Great Ormond Street Hospital Biomedical Research Centre Great Ormond Street Institute of Child Health, UCL London UK; ^4^ Great Ormond Street Hospital NHS Foundation Trust London UK; ^5^ AP‐HP Sorbonne University, Institute of Myology, AFM‐Telethon, I‐Motion Pediatric Clinical Trials Platform, Armand Trousseau Hospital Paris France; ^6^ Department of Neurology Leiden University Medical Center Leiden the Netherlands; ^7^ The John Walton Muscular Dystrophy Research Centre Newcastle University and Newcastle Hospitals NHS Foundation Trust Newcastle upon Tyne UK; ^8^ NIHR Newcastle Biomedical Research Centre University of Newcastle Newcastle upon Tyne UK; ^9^ Centre de Référence Des Maladies Neuromusculaires, CHU de Liège University of Liège Liège Belgium; ^10^ Department of Paediatrics MDUK Oxford Neuromuscular Centre Oxford UK; ^11^ NIHR Oxford Biomedical Research Centre University of Oxford Oxford UK; ^12^ Institute of Myology Paris France

## Abstract

**Aim:**

To investigate the associations between mutations expected to differentially affect Dp140 expression and long‐term trajectories of respiratory and upper‐limb motor outcomes in Duchenne muscular dystrophy (DMD).

**Method:**

In a retrospective analysis of population‐based longitudinal data from three real‐world and natural history data sources, individuals with DMD aged 5 years to 18 years were subdivided according to the predicted effects of the participants' *DMD* mutation on dystrophin isoform expression (group 1, Dp427 absent, Dp140/Dp71 present; group 2, Dp427/Dp140 absent, Dp71 present).

**Results:**

A total of 459 participants were studied, with upper‐limb outcomes assessed in 71 (27 in group 1 and 44 in group 2) and forced vital capacity percentage predicted (%pred) assessed in 434 (224 in group 1 and 210 in group 2). Mean grip strength %pred was on average 7.1 percentage points lower in group 2 than in group 1 (*p* = 0.03). Mean pinch strength %pred was on average 9.2 percentage points lower in group 2 than in group 1 (*p* = 0.04). Mean forced vital capacity %pred was on average 4.3 percentage points lower in group 2 than in group 1 (*p* = 0.01).

**Interpretation:**

In individuals with DMD, *DMD* mutations predicted to affect Dp140 expression were associated with more severe trajectories of respiratory and upper‐limb motor outcomes.

## Graphical abstract

Mary Chesshyre, Deborah Ridout, Georgia Stimpson, Valeria Ricotti, Silvana De Lucia, Erik H Niks, Volker Straub, Laurent Servais, Jean‐Yves Hogrel, Giovanni Baranello, Adnan Manzur, UK NorthStar Clinical Network and Francesco Muntoni* on behalf of the iMDEX network.

Males with Duchenne muscular dystrophy aged 5 years to 18 years were subdivided according to the predicted effects of the participants' *DMD* mutation on dystrophin isoform expression (group 1, Dp427 absent, Dp140/Dp71 present; group 2, Dp427/Dp140 absent, Dp71 present).

Predicted reduced Dp140 expression was associated with reduced grip and pinch strength.

Predicted reduced Dp140 expression was associated with reduced forced vital capacity percent predicted.

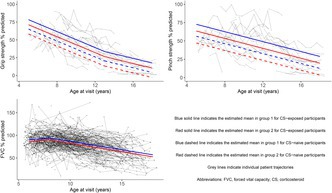

Plain language summary: https://onlinelibrary.wiley.com/doi/10.1111/dmcn.16331

This original article is commented by Birnkrant on pages 1241–1242 of this issue.

**Abbreviations**
%pred: percentage predicted
DMD: Duchenne muscular dystrophy
FVC: forced vital capacity

**What this paper adds**

- Predicted reduced Dp140 expression was associated with worse Duchenne muscular dystrophy outcomes as evidenced by reduced grip and pinch strength.
- Reduced forced vital capacity percentage predicted was also observed.

Duchenne muscular dystrophy (DMD) is caused by *DMD* mutations leading to absent or severely deficient levels of dystrophin protein.^1^ This results in progressive muscle weakness, progressing to loss of ambulation, respiratory insufficiency, and cardiomyopathy.^1^, ^2^ DMD is also associated with intellectual disability, autism spectrum disorder, attention‐deficit/hyperactivity disorder, and anxiety.^3^, ^4^, ^5^, ^6^, ^7^, ^8^

There is considerable heterogeneity in the DMD clinical course.^9^ Some heterogeneity has been associated with *DMD* mutations allowing for the production of low levels of dystrophin via endogenous exon skipping.^10^, ^11^, ^12^, ^13^, ^14^ Another fraction of heterogeneity has been associated with changes in genes other than *DMD* (gene modifiers).^14^, ^15^, ^16^, ^17^, ^18^, ^19^ However, this leaves a large portion of the underlying causes of this heterogeneity not understood.

The *DMD* gene produces several dystrophin isoforms with different sizes and tissue‐specific expression patterns. The full‐length isoform Dp427 exists in three variations: Dp427m, Dp427c, and Dp427p. The isoform predominantly expressed in skeletal muscle is Dp427m.^20^ Dp427m, Dp427c, and Dp427p are expressed at low levels in the human brain.^21^, ^22^ The shorter isoform Dp140 is expressed in the human brain and developing kidney.^21^, ^23^, ^24^, ^25^

While all *DMD* mutations causing DMD inevitably disrupt Dp427 expression, the *DMD* mutation can also disrupt Dp140 expression depending on its location along the gene.^26^ Dp140 is driven by promoters downstream from the Dp427 promoter.^26^ Previous studies showed an association between loss of Dp140 and Dp71 and higher rates of intellectual impairment in DMD with a cumulative effect of loss of isoforms.^5^, ^27^, ^28^, ^29^

We previously observed that individuals with DMD lacking Dp140 have a more severe motor phenotype.^26^ Individuals with DMD were subdivided according to expected patterns of dystrophin isoform expression, including group 1 (Dp427 absent, Dp140/Dp71 present) and group 2 (Dp427/Dp140 absent, Dp71 present).^26^ The North Star Ambulatory Assessment is a 17‐item DMD‐specific scale of motor function.^9^ Mean peak North Star Ambulatory Assessment scores were lower in group 2 than in group 1.^26^

To further explore these findings, we hypothesized that individuals with DMD with mutations expected to differentially affect Dp140 expression, in addition to Dp427, may have different patterns of respiratory and upper‐limb motor involvement compared to those expected to express Dp140. We evaluated relationships between expected patterns of Dp427 and Dp140 expression and respiratory and upper‐limb motor outcomes in a large cohort of young males with DMD from three multicentre studies. We focused on the respiratory trajectory as measured by trajectories of forced vital capacity (FVC) percentage predicted (%pred), and three upper‐limb motor outcomes: grip strength %pred, pinch strength %pred, and MoviPlate scores.

## METHOD

### Study design

FVC %pred data were gathered from the Outcome Measures in DMD: A Natural History Study (NCT02780492) and the UK NorthStar Clinical Network. Grip strength, pinch strength, and MoviPlate data were gathered from two natural history studies (NCT02780492 and NCT00993161).

NCT02780492 was an international, multicentre, prospective, longitudinal, natural history study involving five European centres: London (UCL Great Ormond Street Institute of Child Health); Newcastle upon Tyne (John Walton Muscular Dystrophy Research Centre); Paris (Institute of Myology); Leiden (Leiden University Medical Center); and Nijmegen (Radboud University Medical Center). The study was registered with the ClinicalTrials.gov website (NCT02780492). The study protocol, and consent and assent documents, were approved by the ethical review boards at the participating institutions. Written informed consent was obtained from study participants and/or their parent/legal guardian. Patients with severe intellectual impairment, who would be unable to cooperate with the examination, was an exclusion criterion for this study. The data included from this study cover the time from 27th June 2012 to 27th October 2020.

The UK NorthStar Clinical Network (see Acknowledgements for the list of centres) is a network of UK neuromuscular centres looking after patients with DMD.^9^, ^30^ Clinical data are collected after obtaining written informed consent. The project has Caldicott Guardian approval and assessments are conducted according to the principles of the 2000 Declaration of Helsinki and its later amendments. According to Caldicott guidelines, only anonymized data are used for analysis. There are no specific exclusion criteria to participation in the UK NorthStar Clinical Network based on cognitive function. The data included from this study cover the time period from 12th September 2005 to 3rd April 2023.

The Upper Limb Evaluation in Non Ambulatory Patients With Neuromuscular Disorder study was a prospective, multicentre, longitudinal, natural history study in non‐ambulant participants with DMD registered with the ClinicalTrials.gov website (NCT00993161); it took place between January 2010 and January 2013.^31^ Patients from the following neuromuscular centres were invited to participate in the study: France (Institute of Myology, Trousseau Hospital, Necker Hospital, Paris; Raymond Poincaré Hospital, Garches; Swynghedauw Hospital, Lille) and Belgium (University Hospital, Gent; CHR La Citadelle, Liège).^31^ The study was approved by the French Ethics Review Board Paris VI (registration no. 2009‐A00600‐57) and the Belgian Ethics Review Board of Gent and Liège; written informed consent was provided by all participants or the parents of minors.^31^ Patients with severe cognitive impairment, limiting their understanding of the tasks to be performed, was an exclusion criterion for this study. The data from this study were collected between 4th March 2010 and 9th January 2013.

### Testing procedures

Upper‐limb strength was tested by determining grip strength using the MyoGrip dynamometer and pinch strength using the MyoPinch dynamometer.^32^ The MyoGrip dynamometer is an electronic device that measures grip strength; the MyoPinch dynamometer measures key pinch.^32^ Upper‐limb function was tested using the MoviPlate device, which measures the ability to produce repeated hand and finger movements between two cylindrical target keys by pressing each target alternately as many times as possible in 30 seconds and counting back and forth taps.^32^

### Participant characteristics

We included participants with DMD aged from 5 years to 18 years for the upper‐limb cohort. Participants were included if aged from 6 years to 18 years for the respiratory cohort because of the reduced reliability of spirometry below the age of 6 years. Included participants had a *DMD* mutation that was predicted to be out of frame and/or frameshift and/or a nonsense mutation. Participants were grouped into two groups based on the predicted *DMD* mutation effect on dystrophin isoform expression as follows: group 1 (Dp427 absent, Dp140/Dp71 present) and group 2 (Dp427/Dp140 absent, Dp71 present). Group 1 consisted of participants with *DMD* mutations involving only the genomic region upstream of intron 44; group 2 consisted of participants with *DMD* mutations involving the region from exon 51 to exon 62 inclusive, but not the region of exon 63 or downstream of exon 63.^26^ Participants with *DMD* mutations predicted to result in loss of Dp427, Dp140, and Dp71 were excluded from the analysis because of the small number of patients with data available in this subgroup. Participants with *DMD* mutations involving exons 45 to 50 inclusive and not involving the genomic region of exon 51 or downstream of exon 51 were excluded because it is difficult to determine the effects of these mutations on Dp140 expression.^26^, ^33^

### Statistical analysis

Population characteristics are presented as the mean and standard deviation (SD) for continuous data and frequency or percentage for categorical data.

For the outcomes grip strength %pred, pinch strength %pred, and MoviPlate scores, mixed effects regression models were fitted, which account for the longitudinal nature of the data and repeated measures from the same patient, to explore the relationship with age and investigate the impact of isoform group on outcomes. For the outcomes grip strength %pred, pinch strength %pred, and MoviPlate scores, age at the visit, isoform group, and corticosteroid exposure group were included as fixed factors and patient was included as a random factor. Model fit was assessed using the Akaike information criterion and the Bayesian information criterion. Interactions between the isoform group and age were considered to allow for differences in slopes between isoform groups; however, these did not improve the model fit in all cases. Where the relationship between the outcome and age was not linear, we used a piecewise linear spline with a knot at age 13 years to model the change in relationship with age. This value of 13 years was used because previous studies demonstrated a different trajectory for grip strength in ambulant and non‐ambulant young males with DMD and visual inspection of our data with locally estimated scatterplot smoothing showed a change in the relationships with age of MoviPlate scores and grip strength after around 13 years of age.^12^, ^34^, ^35^ For pinch strength %pred, there was no evidence that the relationship was non‐linear. Models were adjusted for corticosteroid use (corticosteroid‐naive or corticosteroid‐exposed).

For the FVC %pred outcome, a mixed effects regression model was fitted. For the outcome of FVC %pred, age at the visit and isoform group were included as fixed factors and patient was included as a random factor. This accounts for the longitudinal nature of the data. The corticosteroid exposure group was not included as a fixed factor for the outcome of FVC %pred because all participants included in the analysis of FVC %pred were corticosteroid‐exposed. This accounts for the longitudinal nature of the data. A piecewise linear spline was used to model non‐linearity and a knot at 8 years, based on exploration of the data and trajectories reported in previous studies.^13^, ^14^ Models were compared using the Akaike information criterion and the Bayesian information criterion.

Estimates are presented as mean yearly change for age and estimated difference in mean between isoform groups and between corticosteroid groups, with 95% confidence intervals; *p <* 0.05 was deemed statistically significant. All statistical analysis was carried out and plots were generated using RStudio v4.2.2 (31st October 2022) (Posit PBC, Boston, MA, USA).

## RESULTS

### Clinical and genetic features of the study population

Clinical data from 459 participants were included (388 participants were only in the respiratory cohort, 25 participants were only in the upper‐limb cohort, and 46 participants were in both the respiratory and upper‐limb cohorts); these are summarized in Table 1. The characteristics of the study population according to the original data source are outlined in Table 2.

**TABLE 1:** Clinical and genetic features of the study population.

	Upper limb			Respiratory		
	Overall	Group 1	Group 2	Overall	Group 1	Group 2
Number of participants	71	27	44	434	224	210
Age range in years:months (min–max)	5:0–18:6	6:3–18:5	5:0–18:6	5:6–18:5	5:7–18:4	5:6–18:5
Age at first visit (years:months), mean (SD)	11:1 (3:10)	12:0 (3:4)	10:5 (4:0)	8:7 (2:6)	8:6 (2:4)	8:8 (2:9)
Age at last visit (years:months), mean (SD)	13:1 (3:8)	13:9 (2:11)	12:8 (4:0)	11:3 (3:4)	11:5 (3:3)	11:1 (2:5)
Number (%) CS‐naive	14 (19.7)	10 (37)	4 (9.1)	0 (0)	0 (0)	0 (0)
Number (%) CS‐exposed	57 (80.3)	17 (63)	40 (90.9)	434 (100)	224 (100)	210 (100)
Total number of visits	332	112	220	1529	819	710
Mean number of visits per participant	4.7	4.1	5	3.5	3.7	3.4
Duration of follow‐up (years:months), mean (SD)	2:0 (1:5)	1:9 (1:2)	2:2 (1:6)	2:8 (2:9)	2:11 (2:9)	2:5 (2:9)

*Note*: Of the 332 visits for the upper‐limb cohort, grip strength percentage predicted data were included for 318 visits, pinch strength percentage predicted data were included for 321 visits, and MoviPlate data were included for 316 visits. For participants from the respiratory cohort, for whom data were included from both the UK NorthStar Clinical Network and NCT02780492, the follow‐up duration used was the time between the first and last visit for participants; however, this may not be a continuous period of follow‐up because these participants may have had a gap in time between participating in the two studies. This is broken down according to study in Table 2. Abbreviations: CS, corticosteroid; NCT, ClinicalTrials.gov identifier.

**TABLE 2:** Characteristics of the study population according to the original data source.

Characteristic	Upper limb		Respiratory		
	Study NCT02780492	Study NCT00993161	Study NCT02780492 only	NS only	NS and study NCT02780492
Number of participants	53	18	29	391	14
Total number of visits	285	47	124	1316	89
Mean number of visits per participant	5.4	2.6	4.3	3.4	6.4
Duration of follow‐up (years:months), mean (SD)	2:5 (1:5)	0:11 (0:4)	2:2 (1:9)	2:7 (2:9)	6:3 (2:11)^a^

Abbreviations: NCT, ClinicalTrials.gov identifier; NS, UK NorthStar Clinical Network.

^a^ Participants from the respiratory cohort for whom data were included from both the UK NorthStar Clinical Network and study NCT02780492; the follow‐up duration in Table 2 is the time between the first and last visit for these participants; however, this may not be a continuous period of follow‐up because these participants may have had a gap in time between participating in the two studies. Forty‐six participants were in both the respiratory and upper‐limb cohorts; all were from study NCT02780492.

### Relationships between dystrophin isoform group and upper‐limb motor outcomes

**FIGURE 1:**
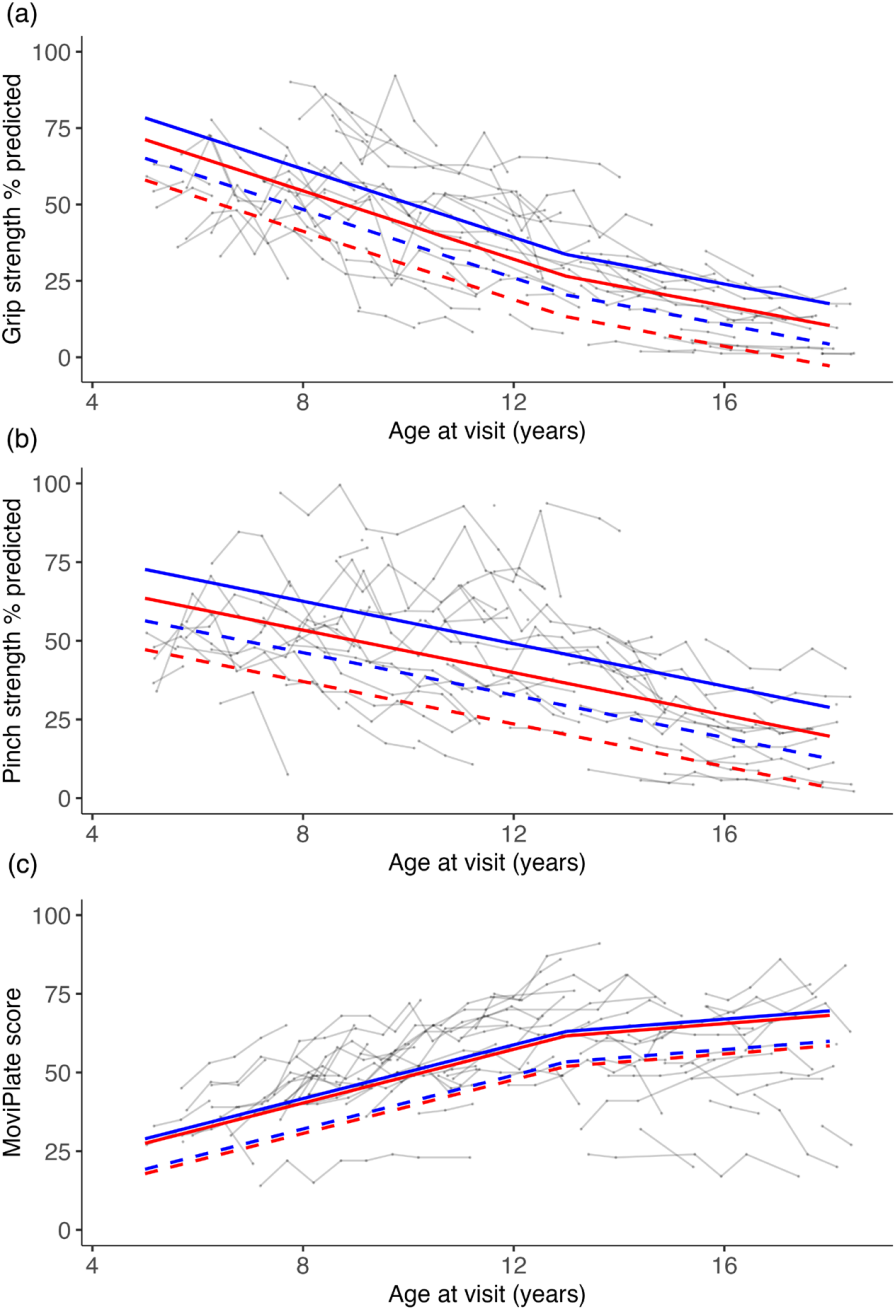
Slopes of yearly progression in grip and pinch strength percentage predicted and MoviPlate score according to the isoform and corticosteroid group. The blue solid line indicates the estimated mean in group 1 for corticosteroid‐exposed participants; the red solid line indicates the estimated mean in group 2 for corticosteroid‐exposed participants; the blue dashed line indicates the estimated mean in group 1 for corticosteroid‐naive participants; the red dashed line indicates the estimated mean in group 2 for corticosteroid‐naive participants; the grey lines indicate individual patient trajectories.

Mean grip strength %pred showed a mean decline of 5.3 percentage points per year (*p* < 0.001) in those aged 5 years to 12 years and a mean decline of 2.9 percentage points per year (*p* < 0.01) in those aged 13 years to 18 years (Table 3 and Figure 1). Mean pinch strength %pred showed a mean decline of 3.1 percentage points per year (*p* < 0.001) in those aged 5 years to 18 years. MoviPlate scores showed a mean improvement of 4 points per year (*p* < 0.001) in those aged 5 years to 12 years and little annual change in those aged 13 years to 18 years (mean increase of 1.2 points per year, *p* = 0.11).

**TABLE 3:** Relationships between isoform group and upper‐limb motor outcomes.

	Parameter	Estimate (95% CI)	*p*
Grip strength %pred	Annual change in those aged 5–12 years	−5.3 (−6.2 to −4.4)	**< 0.001**
	Annual change in those aged 13–18 years	−2.9 (−4.7 to −1.1)	**< 0.01**
	Difference in mean grip strength %pred between group 2 and group 1 across all age points	−7.1 (−13.7 to −0.6)	**0.03**
	Difference in mean grip strength %pred between CS‐exposed and CS‐naive groups across all age points	13.2 (5.4 to 21.1)	**< 0.01**
Pinch strength %pred	Annual change in those aged 5–18 years	−3.1 (−3.7 to −2.6)	**< 0.001**
	Difference in mean pinch strength %pred between group 2 and group 1 across all age points	−9.2 (−17.8 to −0.5)	**0.04**
	Difference in mean pinch strength %pred between CS‐exposed and CS‐naive groups across all age points	16.3 (5.6 to 27.1)	**< 0.01**
MoviPlate scores	Annual change in those aged 5–12 years	4 (3.3 to 4.7)	**< 0.001**
	Annual change in those aged 13–18 years	1.2 (−0.3 to 2.7)	0.11
	Difference in mean MoviPlate scores between group 2 and group 1 across all age points	−1.4 (−7.1 to 4.2)	0.62
	Difference in mean MoviPlate scores between CS‐exposed and CS‐naive groups across all age points	9.7 (0.5 to 18.8)	**0.04**

*Note*: Estimates for differences between isoform groups were adjusted for age and corticosteroid (CS) exposure. Significant *p*‐values are highlighted in bold.

Abbreviations: %pred, percentage predicted; CI, confidence interval.

Group 2 participants had lower mean grip strength %pred than those in group 1 by 7.1 percentage points across all age points (*p* = 0.03). Group 2 participants had lower mean pinch strength %pred than those in group 1 by 9.2 percentage points across all age points (*p* = 0.04). Mean MoviPlate scores did not differ significantly between isoform groups.

Corticosteroid‐exposed participants had higher mean grip strength %pred than corticosteroid‐naive participants by 13.2 percentage points across all age points (*p <* 0.01). Corticosteroid‐exposed participants had higher mean pinch strength %pred than corticosteroid‐naive participants (difference of 16.3 percentage points across all age points, *p <* 0.01). Corticosteroid‐exposed participants had higher mean MoviPlate scores than corticosteroid‐naive participants by 9.7 points across all age points (*p* = 0.04).

### Relationships between dystrophin isoform group and respiratory progression

**FIGURE 2:**
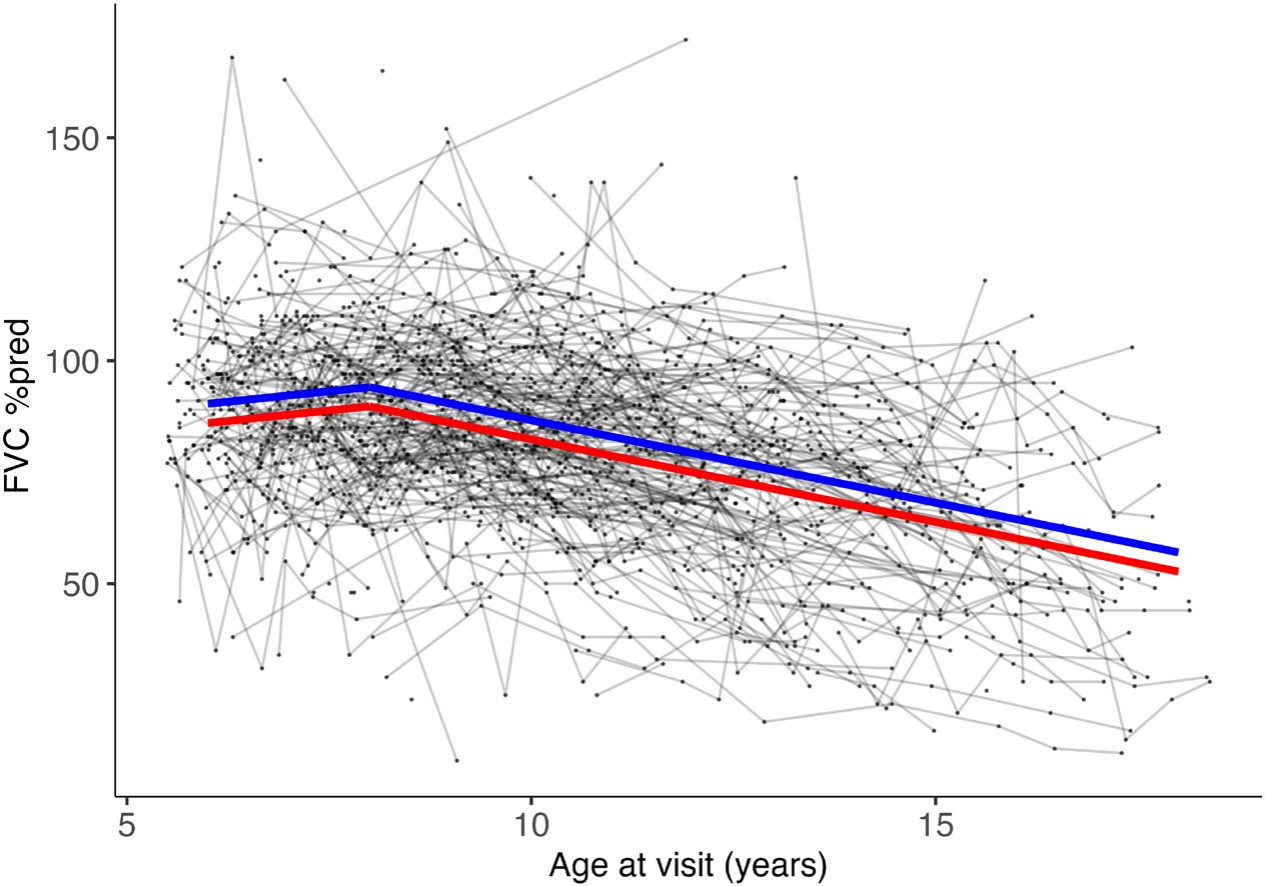
Slopes of yearly respiratory progression according to isoform group. The blue line indicates the estimated mean in group 1; the red line indicates the estimated mean in group 2; the grey lines indicate the individual patient trajectories. All participants included in the respiratory cohort were corticosteroid‐exposed. Abbreviations: %pred, percentage predicted; FVC, forced vital capacity.

Mean FVC %pred showed little annual change in those aged 6 years to 7 years and a mean decline of 3.5 percentage points per year (*p <* 0.01) in those aged 8 years to 18 years (Table 4 and Figure 2). Group 2 participants had lower mean FVC %pred than group 1 participants by 4.3 percentage points across all age points (*p* = 0.01).

**TABLE 4:** Relationships between the isoform group and respiratory progression.

Parameter	Estimate (95% CI)	*p*
Annual change in FVC %pred in those aged 6–7 years	1.5 (−0.1 to 3.1)	0.07
Annual change in FVC %pred in those aged 8–18 years	−3.5 (−5.9 to −1.1)	**< 0.01**
Difference in mean FVC %pred between group 2 and group 1 across all age points	−4.3 (−7.8 to −0.9)	**0.01**

*Note*: Significant *p*‐values are highlighted in bold. All participants included in the respiratory cohort were corticosteroid‐exposed. Abbreviations: %pred, percentage predicted; CI, confidence interval; FVC, forced vital capacity.

## DISCUSSION

In this study, we evaluated the relationships between respiratory and upper‐limb motor trajectories and expected patterns of dystrophin isoform expression in DMD.

To the best of our knowledge, this is the first study to demonstrate associations between predicted lack of Dp140 and lower mean grip and pinch strength in humans with DMD. Our group previously demonstrated lower grip strength in *mdx52* mice (lacking Dp140) compared to *mdx* mice (not lacking Dp140).^26^

A possible explanation for the association between worse grip and pinch strength and expected disruption of Dp140 expression in our study is that young males with DMD lacking Dp140 are more likely to have executive function deficits affecting their ability to plan and carry out the tasks of using the MyoGrip and MyoPinch dynamometers. A study demonstrated deficits in aspects of executive function needed to plan goal‐oriented behaviour and tasks in DMD.^36^ Another study found lower processing speed in males with disrupted Dp140 expression compared to males with intact Dp140 expression.^37^ In another study, Dp140‐negative participants with DMD performed worse in measures of executive function and verbal memory than Dp140‐positive participants with DMD.^38^ The use of wearable devices monitoring movements in real‐life may help delineate the extent to which understanding a task contributes to differences noted between isoform groups.^39^ Another possible explanation for our observed association between predicted interruption of Dp140 expression and lower mean grip and pinch strength is that it is partly related to dysfunction in the cerebellum and cerebellar cortical circuitry. Several studies suggested a role of deficits in the cerebellum and cerebellar cortical connections in DMD central nervous system dysfunction.^36^ In a study of *mdx* mice, in which both brain and muscle full‐length dystrophin isoforms were absent, the absence of dystrophin was associated with altered cerebellar Purkinje cell firing; cerebellar long‐term depression was altered in alert *mdx* mice.^40^ Dp140 is expressed in the human brain at higher levels in the cerebellum than in the cortex.^21^ The cerebellum has an important role in the timing and coordination of grip.^41^ Patients with cerebellar disorders show deficits of predictive grip force control.^41^ A study used functional magnetic resonance imaging (MRI) to investigate patterns of blood‐oxygenation‐level‐dependent activation in the cerebellum in relation to the amplitude and rate of pinch‐grip force production in humans.^42^ They demonstrated blood‐oxygenation‐level‐dependent activation in superior and medial areas of the cerebellum in relation to production of pinch‐grip force at different amplitudes, and in the inferior and lateral areas of the cerebellum in relation to the production of different pinch‐grip force at different rates.^42^ However, we did not find a significant association between the expected disruption of Dp140 expression and MoviPlate scores in this study. Further research is needed to evaluate these hypotheses.

We found that group 2 participants had lower mean FVC %pred than group 1 participants. A study demonstrated associations between FVC %pred and Full‐scale, Performance, and Verbal intelligence quotients in DMD.^43^ Another study found that *DMD* mutations situated at the 3′ untranslated region of *DMD* intron 44 were associated with approximately 6% lower pulmonary function test values in DMD.^14^ A possible explanation for the association between worse FVC %pred and expected disruption of Dp140 expression in our study is that young males with DMD lacking Dp140 are more likely to have executive function deficits affecting their ability to plan and carry out spirometry. This raises the question of whether alternative respiratory function testing, less dependent on understanding and executive function, is required for cognitively impaired young males with DMD. This has important implications for both clinical practice and clinical trials using respiratory outcome measures.

The possibility that a lack of Dp140 affecting motoneuron function contributes to our findings cannot be excluded; however, further research is needed to evaluate this.^26^

Several studies explored the potential effect that chronic corticosteroid exposure might have on brain morphology; however, we did not measure this.^44^, ^45^ A study obtained T1‐weighted MRI from three groups of participants aged 9 years to 18 years, that is, participants with DMD treated with daily deflazacort, participants with DMD treated intermittently with prednisone, and typically developing controls.^44^ The deflazacort group, but not the prednisone group, showed significant differences in grey matter, white matter, and cerebrospinal fluid volumes compared to the control group, after correction for intracranial volume.^44^ In a different MRI study with participants aged between 9 years and 20 years, the daily deflazacort‐treated group with DMD showed differences in subcortical volumes and different patterns of cortical thickness, sulcal depth, and gyrification compared to the intermittent prednisone‐treated group.^45^ More work is required to establish a clear link between steroid exposure and brain development and cognitive function in DMD.

Strengths of our study include the longitudinal nature of data collection and the large number of observations per patient, the prospective nature of the data collected, and the international multicentre data set. Limitations of our study include different lengths of follow‐up in different data sources and missing data. Other *DMD* mutations of interest and changes in genes other than *DMD* (gene modifiers) not assessed in this study may also contribute to genotype effects in the outcomes studied. FVC %pred measurements were collected using different equipment at different sites and there was no centralized monitoring of the technique for collecting FVC %pred.

In summary, our study demonstrates associations between expected lack of Dp140 and lower mean grip strength, pinch strength, and FVC %pred in individuals with DMD.

Further research is needed to evaluate the potential mechanisms underpinning the complex relationships between central nervous system involvement and motor trajectories in DMD.

## CONFLICT OF INTEREST STATEMENT

Mary Chesshyre, Deborah Ridout, Georgia Stimpson, Silvana De Lucia, and Adnan Manzur report no conflicts of interest.

Erik Niks has been a participant in advisory boards for Edgewise, Italfarmaco, Sarepta Therapeutics, Epirium, Regenxbio, and Janssen. Reimbursements were received by the Leiden University Medical Center. He has also worked as Principal Investigator at the Leiden University Medical Center for clinical trials related to muscular dystrophies from Edgewise, Italfarmaco, Sarepta Therapeutics, Fibrogen, NS Pharma, Reveragen, Santhera, BioMarin, and ML Bio.

Jean‐Yves Hogrel is a coinventor of the MyoGrip, MyoPinch, and MoviPlate devices.

Laurent Servais has received consulting fees from Roche, Biogen, Avexis, Cytokinetics, Sarepta Therapeutics, Biomarin, Pfizer, Santhera, Servier, Biophytis, Audentes, Affinia, BioHaven, Scholar Rock, Dyne, Sysnav, PTC, and Dynacure. He conducts research (newborn screening) funded by Roche, Novartis and Biogen. He is a coinventor of the MoviPlate device.

Volker Straub has served on advisory boards for Astellas Gene Therapies, Biogen, Edgewise Therapeutics, Ipsen, Kate Therapeutics, ML Bio Solutions, Novartis Gene Therapies, PepGen, Roche, Sanofi, Sarepta Therapeutics, Vertex Pharmaceuticals, and Wave Therapeutics. He has received speaking fees or honoraria from Novartis Gene Therapies, Pfizer, Roche, Sanofi, and Sarepta Therapeutics; he has received grants for clinical research from Sarepta Therapeutics and Sanofi.

Valeria Ricotti is cofounder of DiNAQOR, Parterra Limited, Salanar Limited, and Vesalic Limited; in the past, she acted as consultant for Solid Bioscience and Antisense Therapeutics.

Giovanni Baranello is Principal Investigator of clinical trials sponsored by Roche, Novartis, Sarepta Therapeutics, Pfizer, NS Pharma, Reveragen, Percheron, Biomarin, and Scholar Rock; he has received speaker or consulting fees from Sarepta Therapeutics, PTC Therapeutics, Pfizer, Biogen, Novartis Gene Therapies (AveXis), and Roche, and grants from Sarepta Therapeutics, Roche, and Novartis Gene Therapies. UCL has received funding from Sarepta Therapeutics, Roche, Pfizer, Italfarmaco, and Santhera.

Francesco Muntoni reports research funding from Sarepta Therapeutics, and participation in scientific advisory boards or clinical trial monitoring groups for Sarepta Therapeutics, Dyne Therapeutics, Dyne, Pfizer, Italfarmaco, and Santhera.

## Data Availability

The data that support the findings of this study are available from the corresponding author (Francesco Muntoni) upon reasonable request.
